# Functional genetic polymorphisms in ILT3 are associated with decreased surface expression on dendritic cells and increased serum cytokines in lupus patients

**DOI:** 10.1186/ar3997

**Published:** 2012-09-27

**Authors:** MA Jensen, KC Patterson, AA Kumar, M Kumabe, BS Franek, TB Niewold

**Affiliations:** 1 Section of Rheumatology and Gwen Knapp Center for Lupus and Immunology Research, University of Chicago, IL, USA

## Background

Hyperactivity of the type I interferon (IFN) pathway is involved in the pathogenesis of systemic lupus erythematosus (SLE). Immunoglobulin-like transcript (ILT3) is an immunohibitory transmembrane molecule that is induced by type I IFNs. ILT3 is expressed by plasmacytoid dendritic cells (PDCs), monocytoid dendritic cells (MDCs), and monocytes/macrophages. Given the pathogenic role of IFN in SLE, we hypothesized that the IFN-induced immunosuppressive ILT3 receptor may be dysfunctional in human SLE.

## Methods

In total, 132 European-derived and 79 Hispanic-American SLE patients were genotyped for two coding-change SNPs predicted to interfere with protein folding in ILT3 (rs11540761 and rs1048801). One hundred and sixteen control DNA samples and sera from healthy controls were also studied. We detected associations between ILT3 genotype and serum cytokine profiles. ILT3 expression levels on PDCs and MDCs from 18 patients and 10 controls were studied by flow cytometry.

## Results

The rs11540761 SNP in the extracellular region was associated with decreased cell surface expression of ILT3 on circulating MDCs and to a lesser extent PDCs in SLE patients. The cytoplasmically located rs1048801 SNP was not associated with a change in DC expression of ILT3. Both SNPs were significantly and independently associated with increased levels of serum type I IFN activity in SLE patients. The rs1048801 SNP was also associated with increased serum levels of TNFα.

## Conclusion

Loss-of-function polymorphisms in ILT3 are associated with increased inflammatory cytokine levels in SLE, supporting a biological role for ILT3 in SLE.

